# The Beginnings of Education

**Published:** 1915-10

**Authors:** Henry Jones Mulford

**Affiliations:** Buffalo, N. Y.


### The Beginnings of Education.

### Some Suggestions to Parents and Teachers.

By HENRY JONES MULFORD, M. D., Buffalo, N. Y.

### Third Paper—The Result.

The aim of education is the development of self-consciousness; the individual must know what he knows. If his knowledge is not at his command then he is not educated. In order to be at his command his knowledge must be in his conscious memory, not merely in his reflex memory. If it is reflex he has no control over it, he cannot himself call it into action, its activity may be aroused only through some external stimulus. That is the difficulty with most educations, they are reflex; that is, the individual is not aware of the extent of his own knowledge; he has no command over himself. That is why so many of us are so far removed from the ideal. The ideal individual should be the sum of his brain centres, his centres should work together in an evenly balanced harmony, each centre having developed its conscious memory in the proper manner. But, the individual of today does not ap proach the ideal in that respect. In fact, there is no present ideal individual. Mr. James Huneker, in a paper on "The Music of Today and Tomorrow," in the Century Magazine for May, 1914, approaches the truth when he says: "Medi·ocrity is mankind in the normal." But the statement is not true, for, mediocrity is **not** the normal. It may seem to be to the superficial observer, but the **actual** normal for mankind is the **ideal**; that is, the sum of the brain centres. What Mr. Huneker should have said is, that mediocrity is mankind in the **average,** for that, alas! is the truth. The brain centres of the everyday individual have not developed uniformly; one can say, almost, that they have not seemed to develop at all. They have grown as anatomical units, but they have not grown as thought units: the individual has not learned to use the anatomical units. And so, as the brain centres **are** the individual, one readily can understand why the average is mediocrity.

In our several discussions we have uncovered two basic facts: that the critical period in the growth of the human brain is during the time of its mass development, during the first eight years of its life; and, that the development of the cells of the cortex of the human brain depends upon memory, upon the capacity of those cells for storing up sensory impressions. But, now, there is another fact of equal importance, which may be put in these words: That every human brain carries within its cells the menace of self-impairment through the outcropping of a bias.

This phrase, "the outcropping of a bias," harks back to the fact that the brain centres are developed by what goes into them; that that which goes in goes into the memory, and, remaining there, is liable at any moment to assert itself. To be exact it means this: that some portion of the cerebral cortex may become overactive and dominate the rest, completely altering the outlook of the individual. Knowing this we can understand how great a menace the past of an individual may become. If the individual is careless as to what goes into his memory he is going to reap the result of his carelessness. This should be impressed upon the mind of the individual: every child should know that each **present** thought, each idea, has a future. In this connection we recall the words of Banquo to the witches in "Macbeth."

"If you can look into the seeds of time,
And say which grain will grow, and which will not."

The seeds of time! What are they? What, indeed, but our thoughts, our words, our actions? But, after all, one does not have to be a witch to "look into the seeds of time;" one does not have to be a witch to look into his own thoughts, into his own actions. These are the things that make the future; these are the things that go to upbuild the brain structure. But the individual must know how to build; he must know how to select his materials, what to take and what to avoid. There must be no had spots in the "walls" of this structure, for bad spots mean weakness. In a structure built of stone or brick a bad spot means actual weakness of structure; but in the structure made up of brain cells the bad spots, **the bias,** means strength, and this kind of strength is bad for the individual. We often hear it said of a person who has gone wrong in some particular that he was weak in that direction; but that is not the truth. It is not weakness that causes a man to go wrong, it is strength. **Weakness never leads.** A man's character is determined by his strongest attributes, those attributes that lead and overshadow the others.

It is the easiest thing in the world for an idea to secure a foothold within the brain and later to develope a bias. The brain is so open to every passing influence that it is almost impossible to supervise all that goes into it. This being so the question arises, is there a remedy? There is, although the difficulties of administering it are many. Remembering that human brain development has come through action we must proceed with the building up of our brain structure with that in mind. If our beginning is right and we develope the child brain as it should be devedoped we early bring that brain to a realization of its own position and power; we develop the child's self-consciousness early. We must bring the child to understand that he is to direct the activities of his brain centres, not that he is to allow the centres to direct him. **He must not allow his brain centres to dominate.** He is to use his inner vision, his reason, and by it to regulate and to harmonize the action of his brain centres. Dominance by his centres would mean anarchy for him, for then the action of the centres becomes haphazard action; that is, action without conscious direction. **It is this action that is behind the development of the bias.**

Brain habits that come through conscious direction are habits that are formed in the proper manner, and, are, therefor, good habits; habits that come unconsciously, that form themselves, are habits that are formed in an improper manner, and, are, therefore, bad habits. Bad habits develop under poor teaching, or where there is no teaching at all. In either case this means that the child himself has had the greatest influence upon himself; the greatest influence, and, under the circumstances, the worst influence. If this has gone on long enough there have been some parts of the brain structure begun, some portions of its "walls" have been laid, or, some "stones" have been set irregularly here and there. In both cases the bias is a very real danger; and, it is in these cases that the later education is apt to become just a veneer, a veneer that but thinly covers the baser material underneath. When this veneer warps and cracks the bias shows forth.

In the child who has had no regular teaching and who has begun the formation of bad brain habits without knowing it there has not been a great deal of harm done because the sensory impressions coming in to the brain cells have been more or less scattered; while in the child who has had bad teaching the impressions have been directed in some sort of order, even though the order has been a wrong one, and the impressions have become more or less permanent. In the first there has been no real beginning; in the second there has been a wrong beginning. And, so, it is not difficult to understand that the no-beginning child will be easier to manage than the wrong-beginning child. In the child already under way there is the wrong to overcome; the "walls" that have been begun in this brain structure are obstruction walls, they obstruct his further progress. In the no-beginning child there are no set walls, only an occasional obstructing stone, and, there is not, therefor, so much to overcome. The main difficulty with the last is that these occasional stones, that is, independent ideas, are most often fault-ideas that, later, will develope into biases. And fault-ideas may not be eradicated from the brain any more than any other idea may be eradicated that has secured a place therein. All that may be hoped for is that these spots may be surrounded by good ideas. New, strong, ''walls'' must be built about the old; new, attractive ideas, ideas that form logical sequences, must obliterate the harmful, disjointed fault-ideas. In this connection I wish again to assert my belief that no child under eight years of age should receive formal teaching. Before that age there should be discipline and a preliminary training of the special senses, but there must be no formal teaching in the very young. Formal teaching puts too great a strain upon the immature brain centres, while preliminary training under discipline develops the centres and avoids the implantation of fault-ideas.

As we learned in the first paper of this series, before the age of eight the brain centres are not structurally ready for complete function. The centres early begin to receive sensory impulses, but no centre can respond to these impulses until the cells and the transmitting nerve fibres are ready for concerted action. And, further, before a brain cell can respond to a sensory impulse it must have received a number of the same impulses. In other words, not only must a centre be structurally ready for function, but, it also must have had some preliminary training before it properly can respond to the received impulse. And, so, in the young child, the preliminary training is a training aimed at the development of the cells of the cerebral cortex, a development that comes as a natural thing, without effort on the part of the cells themselves. This training is solely developmental, and, in fact, amounts to what might be called passive exercise of the cells. But this training is the most important thing in the history of the child, for, upon it depends the entire future of the individual. This is the period when the brain is increasing its bulk, and its cells are being developed by what is going into them: it is the time when the sensory impressions grow into the brain substance and become a part of it, as it were. These impressions grow into the memory, and, because they do, they may not be eradicated. They remain either to make or to mar the individual.

The normal baby is born with all his brain centres as potential units; the cells are there ready for development into function. As the baby advances into life, as the infant becomes the child, the centres take up their function as the demand comes to them. As we have seen there is a regular developmental sequence, but no centre comes into active action without a preliminary period of training. Of the three groups of centres the motor group and the special sense group have, as it were, grown up with the brain; they are an indelible part of it. The third group, that of the higher centres, is a later development. The first and second belong to the period of brute development, the third to the period of man development. The period of man development has added, and is adding, to the merely animal centres the man refinements. In the motor centres there is a finer action and a closer co-ordination; in the special sense centres the field of action has been increased. In the change from brute to man, muscle action has progressed from the exhibition of mere brute strength to the fine adjustments of the skilled workman. In the development of the special senses the area of the centres has been increased and the cell development of each centre has been advanced to a finer stage. Take the sense of hearing for example. In the beginning that centre was a centre for the reception of just sound, it was nothing more than a faculty by means of which the individual guided and protected himself. Gradually he learned to differentiate sounds and to locate them, and, from then on, the sense of hearing became a means for the advancement of the animal. In man this centre is taking on a wider and wider area of usefulness. Through the larger development the centre now is able to recognize and to place any sound, whether it be just gross sound or a musical note; through this man has been able to develop innumerable musical instruments, and to recognize the voices of his friends; through it a "word" centre and a language centre have been added to the cerebral cortex. It is important that we remember how this centre has advanced in its development, for it is through the same process that the hearing is developed in the child. The first sound of the word "Mama," for instance, is just sound in the child's ears, and, as such, is referred to the "sound" centre. Later, after a sufficient number of repetitions of the word, it "orients" itself, as it were, and the word centre takes charge of it. The mechanism of this orientation is somewhat complicated, and we can understand, therefore, the necessity for the repeated preliminary word impulses.

And, as it is with the centre for hearing, so is it for each of the other centres. The mechanism is complicated in each, and in each there must be a period of preliminary training through which the mechanism is brought into right working order.

And when must the training of the centres begin? When the individual begins to use them for himself. We have seen that the cells of all the brain centres have a double action: they have an automatic or habit action, and they have conscious action. It is the habit action which we want to overcome, and we do this through the training of the conscious action. If we put consciousness in command all else must become secondary; reflex thought, or habit action of the thought cells, no longer dominates. In order to bring this about we shall have to take the child in hand and show him how to get the most out of his special senses: show him how to use his eyes, his ears and his hands, the other special senses not counting for much in the development process. We must show him where his destiny lies, and, then, how he may achieve that destiny; we must make him aware of his own power. The child's beginning instruction must be in the elementals of everyday things. The elementals of sound, of colors, of form, of words, of everything with which he comes in contact. It is through these things that his future is to come, that his success is to be directed. When he takes command of his brain centres he takes command of himself and the development of his own character. There can be no strength of character without the knowledge to develop that strength; there can be no character unless self-consciousness directs it. It is for this reason that brute animals have no character such as man has. Their actions are merely habit actions, reflex actions. We can teach animals to do certain acts, but we cannot make them think. They do not reason out their actions, they merely repeat them through habit. In support of this contention here is a newspaper clipping. Unfortunately I have not been able to trace this story to any source, but it has the sound of fact, and certainly accords with theory:

> "Curiosity and cowardice," said the one-legged veteran, "are the chief characteristics of all monkeys and of most men. I was keeper of the monkey house in a zoo after the war. My biggest charge was an ape the size of a twelve-year-old boy, and it was through his curiosity and cowardice that I used to manage him. We exercised this ape in the big room every day, but when we wanted him to go back to his cage, he'd climb up to the roof of the room, and, even with food, you could not tempt him down. So I would go to Jack Lover and take him by the arm and direct his attention in a mysterious manner to the dark passage under the steam pipes. We would tip-toe to the pipes and pretend to point out to each other some horrible unknown creature in the passage, and I would say: 'There he is! There he is!' As we bent over to look more closely we'd hear the patter of feet behind us. The ape's curiosity had got the better of him. He came and crouched beside us, and peered, with us, into the dark passage fearfully. Then suddenly Lover would shout: 'Look out! He's coming out—he's coming out!' and we'd scamper away in the direction of the ape's house. But the ape would be ahead of us. He'd rush into his house in a perfect whirlwind of excitement and terror. Then—click—we'd snap the door to on him, and he'd look very foolish. Every day we fooled the ape in this way. He was long, you see, on curiosity and cowardice, but very short on memory."

In special training of the special senses it is **brain** training at which we aim, not merely sense training. The average person uses his special senses only superficially. If he looks at an object he merely glances at it, and, an hour afterward, he is unable to give a clear description of what he has seen. His eyes have seen the object, but his brain has not; that is, his brain has not been impressed by the object—impulse. The object has been seen, but it has not been analyzed as to its peculiarities. Now, no one can analyze who does not know how to analyze; no one can analyze who does not know the component parts of that which he seeks to analyze. And, so, in this training we get down to the component parts, the elementals. These the brain learns, and, through this knowledge, directs the action of the special senses, as well as the action of the other brain centres. When the individual can do this he has become self-conscious; he has become aware of his own powers. But, behind this there must be memory; man would get nowhere without that. The lack of it keeps the brute just a brute. The first consideration, then, in the development of man is the development of the memory.

When the preliminary training of the senses is completed, when the child brain has completed its mass growth, and the time for beginning formal instruction approaches, what is to be the procedure? The procedure is much the same as before. While the individual has been thoroughly grounded in the elementals he is yet immature as to judgment. The brain has attained its full size, but it is immature as to experience. So far as its exprience goes the child brain is capable of forming its own judgments, but, when it approaches new territory, it will need assistance in entering that territory. The new has to be analyzed as had the old, and the child has to learn the elementals of the new before he may proceed. To leave the child to himself, even though the right start has been made, is wrong, for the child, not yet entirely sure of himself, easily may get off the right track. He needs to be assisted for a time. It will be time enough to put the child upon his own resources when we are sure that those resources are ample for his needs. We want him to strengthen his own powers of observation and decision through his own efforts, but we need to watch him to see that he is so well versed in the elementals that his procedures are sound. But, the child's progress will be less and less difficult from now on. If the foundation of his brain structure has been laid aright the stones of the superstructure will fall into place naturally. The child's memory having been developed along right lines his self-consciousness will assert itself more and more as his experience grows, and, at last, will become so strong that the child safely may be left to himself.

In conclusion let me quote a sentence from Winston Churchill's new book, "A Far Country." In the first chapter the principle character is reviewing his youth, and, at the end of the chapter, he utters these significant words: "At all events, when I look back upon the boy I was, I see the beginnings of a real person who fades little by little as manhood arrives and advances, until suddenly I am aware that a stranger has taken his place."

To me that sentence is deeply pathetic. There is high tragedy in the words: "I see the beginnings of a real person who fades little by little as manhood arrives and advances." The real person disappears and an unknown takes his place; the boy grows into the man, but the boy within, **the real person,** is not at home in his **new** surroundings. **The individual does not know himself: the boy has developed into an alien.** That is the result brought about by the modern system of development, but is that the result which we seek? The reader, if he has read these papers upon the education of the young, of which this is the last, may, perhaps, be able to answer that question for himself.

143 Allen Street.

---

**Treatment of Tuberculosis by Compression.** Eugene Grenier of Montreal, Jour. de Med. et de Chir., Aug., gives the following historic notes: Carson of Liverpool, 1822, suggested that pulmonary collapse would expedite healing; Ramagde of London, 1834, arrived at the same idea; Piorry, about 1865, advocated strapping the chest; Forlanini of Pavia began a series of articles on the subject in 1882 but did not do his first therapeutic pneum-thorax operation till 1892, about which time, Murphy of Chicago employed the same measure. Among 327 tuberculous patients observed by Grenier in the year ending June 30, 1915, 19 were selected for artificial pneumo-thorax. 12 cases have been observed long enough for a report. The attempt at pneumothorax failed in one case. Of six cases of complete pneumothorax, 4 have been arrested, 1 ameliorated, 1 has shown no improvement. Of 5 cases of incomplete pneumothorax, good results have been obtained in 2, fair in 1, and failure in 2.

---

**Smokers' Patches.** L. Landouzy, Paris, Jour. de Med. et de Chir., August. Among 164 syphilitics, mainly tertiary, 131, including 4 women showed buccal patches. Of these 81, including 2 women, gave a positive Wassermann. Of 18 syphilitics in another series, 15 presented symmetric, commisural leucoplasia, and of these, 9 gave a positive Wassermann. He considers leucoplasia as valuable a diagnostic sign of syphilis as the Wassermann reaction and syphilis as the essential element in producing the patches, though smoking is the most frequent contributory cause.

